# Synchrotron Radiation Spectroscopy and Transmission Electron Microscopy Techniques to Evaluate TiO_2_ NPs Incorporation, Speciation, and Impact on Root Cells Ultrastructure of *Pisum sativum* L. Plants

**DOI:** 10.3390/nano11040921

**Published:** 2021-04-04

**Authors:** Simonetta Muccifora, Hiram Castillo-Michel, Francesco Barbieri, Lorenza Bellani, Monica Ruffini Castiglione, Carmelina Spanò, Ana E. Pradas del Real, Lucia Giorgetti, Eliana L. Tassi

**Affiliations:** 1 Department of Life Sciences, University of Siena, Via A. Moro 2, 53100 Siena, Italy; simonetta.muccifora@unisi.it (S.M.); barbierifrance88@gmail.com (F.B.); lorenza.bellani@unisi.it (L.B.); 2 European Synchrotron Radiation Facility, Beamline ID21, 71 Av. Rue des Martyrs, 38000 Grenoble, France; hiram.castillo_michel@esrf.fr (H.C.-M.); ana-elena.pradas@esrf.fr (A.E.P.d.R.); 3 Institute of Agricultural Biology and Biotechnology, National Research Council, Via Moruzzi 1, 56124 Pisa, Italy; lucia.giorgetti@ibba.cnr.it; 4 Department of Biology, University of Pisa, Via Ghini 13, 56126 Pisa, Italy; monica.ruffini.castiglione@unipi.it (M.R.C.); carmelina.spano@unipi.it (C.S.); 5 Research Institute on Terrestrial Ecosystems, National Research Council, Via Moruzzi 1, 56124 Pisa, Italy

**Keywords:** agricultural soil, anatase nanoparticles, cell ultrastructure, crystalline phase, micro-XANES, micro-XRF, pea roots, rutile nanoparticles, TEM, titanium dioxide nanoparticles

## Abstract

Biosolids (Bs) for use in agriculture are an important way for introducing and transferring TiO_2_ nanoparticles (NPs) to plants and food chain. Roots of *Pisum sativum* L. plants grown in Bs-amended soils spiked with TiO_2_ 800 mg/kg as rutile NPs, anatase NPs, mixture of both NPs and submicron particles (SMPs) were investigated by Transmission Electron Microscopy (TEM), synchrotron radiation based micro X-ray Fluorescence and micro X-ray Absorption Near-Edge Structure (µXRF/µXANES) and Inductively Coupled Plasma Optical Emission Spectrometry (ICP-OES). TEM analysis showed damages in cells ultrastructure of all treated samples, although a more evident effect was observed with single anatase or rutile NPs treatments. Micro-XRF and TEM evidenced the presence of nano and SMPs mainly in the cortex cells near the rhizodermis. Micro-XRF/micro-XANES analysis revealed anatase, rutile, and ilmenite as the main TiO_2_ polymorphs in the original soil and Bs, and the preferential anatase uptake by the roots. For all treatments Ti concentration in the roots increased by 38–56%, however plants translocation factor (TF) increased mostly with NPs treatment (261–315%) and less with SMPs (about 85%), with respect to control. In addition, all samples showed a limited transfer of TiO_2_ to the shoots (very low TF value). These findings evidenced a potential toxicity of TiO_2_ NPs present in Bs and accumulating in soil, suggesting the necessity of appropriate regulations for the occurrence of NPs in Bs used in agriculture.

## 1. Introduction

Engineered TiO_2_ nanoparticles (NPs) are successfully used in many industrial sectors, rather than bulk material [1,2]. They are present in daily-use products from food industry and pharmaceuticals [3,4], beauty and personal care products [5], in paints [6], fertilizers [7], plant protection products [8], food packaging [9], etc. At the end of products’ lifecycle, TiO_2_ NPs may accumulate in biosolid (Bs) and sewage sludge from municipal wastewater treatment plants (WWTPs), which are largely introduced in agricultural soils and receiving water bodies [10,11]. Indeed, spillover of sewer waters and wastewaters could contribute for TiO_2_ NPs introduction in soils and waters [12].

Titanium dioxide NPs were proven to have both positive and negative effects on plants, so that their benefits to crops as nano fertilizer are receiving increased attention. Recent studies have reported the increase of seed germination in *Brassica napus* [13] and the improved colonization of exogenous plant growth promoting rhizobacteria in a stressed peat soil [14]. Moreover, the application of TiO_2_ NPs to soil increased phyto-availability and uptake of phosphorus by *Lactuca sativa* [15]. In *Arabidospsis thaliana*, TiO_2_ NPs alleviated the toxicity of tetracycline by reducing both oxidative stress and antioxidant enzymes and enhancing plant growth [16]. In tomato fruits, soil amended with sewage sludge containing TiO_2_ NPs increased growth and yield with few changes in the elemental composition and non-altered the biochemical responses [17]. On the other hand, some negative effects were recently reported: in *Pisum sativum* and *Lens culinaris*, oxidative stress [18,19] and imbalance in the mineral nutrition [20]; in wheat crop, altered metabolism [21], in barley, delay in reaching the physiological maturity [22]; in *Allium sativum*, *Lens culinaris* and *Vicia faba*, phyto- and genotoxicity [3,19,23]. Toxicity and benefits of TiO_2_ NPs are thus not only associated with size, but also with concentration [24], and plant species [25] in addition to the properties of receiving soils, which may influence the in-situ TiO_2_ processes and induce changes in NPs’ bioavailability and bioactivity [26,27,28]. Crystalline phase is another variable to be considered, anatase being generally indicated to be more toxic to living organisms [29]. Despite few studies focused on discriminating NPs presence in environmental samples, in vitro cytotoxicity studies, under simulated sunlight irradiation, indicated higher toxicity of rutile NPs when coated with humic acid, probably due to changes on photoactivity [30]. 

The use of biosolids in agriculture is encouraged not only to improve soil quality but also to stimulate the circular economy, promoting recovery and reuse. Considering that 97.6% of human food comes from soil [31], studies in Bs-amended soil became of great environmental significance to understand the effects of nano and larger TiO_2_ particles in real agroecosystems. This importance is endorsed by the debate in Europe on the safety for human health of E171 TiO_2_ food additive, which contain both dimensions particles [32] and by the studies confirming its toxicity on plants [3].

Previous studies showed that the complex system Bs-soil-plant was impacted by the presence of TiO_2_ NPs, inducing negative threats to the quality of both soil and *P. sativum* plants [18,20]. In these studies, the experimental design was conceived to represent real scenarios of best- and worst-case load of NPs (80 and 800 mg TiO_2_ per Kg of soil) through Bs-amended farm soils [18,20]. Modifications in the plant growth, oxidative stress in terms of lipid peroxidation and production of hydrogen peroxide, as well as the reduced bioavailability of some soil mineral nutrients, stress on the soil microbial population and imbalance in the plant mineral nutrition were recorded [18,20]. Although the non-typical dose-effect relationship were observed in these studies, the worst plant response to anatase or mix of anatase and rutile was evidenced [18,20].

Based on these previous results, the present investigation has been planned to give further light on the TiO_2_ NPs entry, speciation, localization, and induced effects in the root cells’ ultrastructure associated with the presence and the accumulation of NPs in a Bs-farming soil system, using image-based techniques. This approach provides important support to identify, localize, and analyze the Ti effects in plants. Synchrotron radiation (SR) based techniques of micro-X-ray fluorescence (μXRF) and micro-X-ray absorption near-edge structure (µXANES) are able to map the preferential localization of metal/metal oxide NPs and spectroscopically identify the crystalline phase or the eventual chemical speciation of the element inside the plant [33,34]. Transmission electron microscopy (TEM) in addition to offering information for size, shape, and aggregation state inside organs or tissues, permits to further visualize the effects of NPs on plant cells’ ultrastructure [3,23].

This study focused on *P. sativum* L. plants grown under controlled and long-term exposure to environmentally relevant TiO_2_ NPs concentration (cumulative load) in a Bs-amended agricultural soil, mimicking as much as possible a real scenario. Titanium dioxide particles’ entry in the root system, bioaccumulation, relative distribution, and localization, as well as the main crystalline form preferentially absorbed and their effect in cells ultrastructure of plant roots were investigated. In particular, the study considered pure anatase and pure rutile phases (separately or mixed) of nano and larger size, tasks still under discussion by their not well-defined effects in crop plants. 

## 2. Materials and Methods

### 2.1. Preparation of Growth Matrices

Soil was collected at the agricultural area of Agri-Environmental Research Center (CiRAA, University of Pisa, Pisa, Italy), while the Bs suitable for soil amendment was obtained in a cake form at a municipal WWTP near Pisa, Italy. Commercial anatase or rutile NPs in a powder form, both with nominal size of 30 nm and purity of 99.9% (US Research Nanomaterials Inc., Huston, TX, USA), and larger TiO_2_ powder particles with nominal size >100 nm, purity of 99.8% (Sigma-Aldrich—Saint Louis, MO, USA), and named here as submicron particles (SMPs) were used. 

The characterization of collected soil, Bs, and the preparation and characterization of the Bs-amended soil were described in detail in our previous study [20]. In short: aqueous suspensions of TiO_2_ particles were prepared by sonication to reach the loading of 800 mg of TiO_2_ per kg of soil in all treatments, thus simulating a long-term span of biosolids in agricultural soil. Firstly, Bs was spiked with the aqueous suspensions and left for 30 days to simulate aging or eventual transformation during wastewater treatment. Non-spiked Bs, prepared with the same procedure using only milli-Q water, was considered as a control. Biosolid slurries were then thoroughly mixed with the soil (sand 93.3%, clay 2.1%, organic matter 1.1%, and pH 7.7) at 3% (dry weight basis, DW) and left to environmental conditions for further reactions with the soil components: In this way, the following growth-matrices were obtained: C (Bs-amended soil control), Ana800 (anatase NPs), Rut800 (rutile NPs), Mix800 (mixture of anatase and rutile NPs, 1:1 ratio) and SMP800 (submicron TiO_2_ particles). 

Hydrated seeds of *P. sativum* L. were sowed in 500 g of each growth matrix in four pot replicates per treatment. *P. sativum* was left to grow in controlled conditions of light (16 h), temperature (16–22 °C), and humidity (near 65%) for 28 days after germination.

### 2.2. Titanium Concentration in Plants

At harvest, roots and aerial parts (leaves + stems) were separated and thoroughly washed. A major portion of the roots was further cleaned by sonication in deionized water to eliminate eventual adhered soil particles and Ti analysis. Biomasses, dried at 40 °C, were acid digested using a two-step digestion method [35] and Ti element was analyzed by inductively-coupled plasma optical emission spectroscopy (IPC-OES, Varian Liberty AX). Titanium distribution in plants was represented by the translocation factor (TF), usually calculated as shoot–root ratio [36], thus representing, at harvest, how much Ti accumulates in the aerial parts in respect to the roots.

### 2.3. Data Acquisition at Synchrotron Facilities

Synchrotron experiments were performed at the ID21 beamline of the European Synchrotron Radiation Facilities (ESRF, Grenoble-Fr) [37] by analysis on powders (soil and Bs) and roots cross-section. The beam was focused to 0.4 × 0.9 µm^2^ with a Kirkpatrick–Baez (KB) mirror system. The energy selection was done using a double crystal fixed exit Kohzu monochromator equipped with Si 111 crystals. The emitted fluorescence signal was detected with energy-dispersive, large area (80 mm^2^) SDD detector equipped with Be window (SGX from RaySpec, High Wycombe, UK). 

Soil and Bs were dispersed in a surface of transparent adhesive Kapton polyimide film mounted on an in-house Cu sample-holder. Similar and adequate portions of root samples from each treatment were cut and cryo-embedded in an OCT (optimal cutting temperature) resin, which were then cryo-sectioned (20 µm) using a Leica cryo-microtome (LN22) and mounted on sample holder immediately after sectioning and maintained below −30 °C until the analysis with synchrotron radiation. 

The ID21 scanning X-ray microscope is equipped with a passively cooled cryogenic system that maintained the samples under cryogenic conditions (−170 °C) for all the course of analysis. Micro-XRF maps were acquired at 5.1 keV with a step size of 1 × 1 µm^2^ and 0.5 × 0.5 µm^2^ 100 ms dwell time. 

Data from µXRF was processed using PyMCA software [38]. First, intensity variations due to synchrotron electron injection were corrected in all raw data. Then, elemental fluorescence intensity peak signals were fitted in PyMCA to obtain elemental distribution maps that were then overlayed as RGB images or temperature and reverse grey intensity maps. Micro-XANES spectra in selected Ti points of interest was measured at the Ti K-edge (4.95 to 5.15 keV energy range, 0.5 eV step and 100 ms dwell time per energy step). Energy calibration was done using a Ti metallic foil and taking the maximum of the first derivative at 4.966 keV. Individual µXANES spectra were processed using Orange software [39] with the Spectroscopy add-on [40]. Measured spectra were normalized using the XAS normalization algorithm included in the spectroscopy pre-processing tools. 

Micro-XANES data were explored with principal component analysis (PCA, see Statistical analysis) to discriminate in all spectra obtained (all samples) the main groups of Ti-containing compounds based on visual spectral features. The four main groups obtained from PCA analysis are named based on the observation of the main spectral features of samples. To perform a more detailed structure analysis, the averaged data from the four groups were exported to Athena software [41]. The spectra were then compared to experimental and theoretical XANES spectra. Experimental reference spectra were obtained from the pure anatase and rutile NPs used to spike the biosolid. Additional experimental Ti spectra from ilmenite and pseudobrookite were obtained from an on-line Ti K-edge XAS database from beamline 10.3.2 at the Advanced Light Source, Berkeley, USA. This database contained a Ti foil reference spectrum, and it was used to match energy calibration to our data. The theoretical XANES spectra were obtained from https://materialsproject.org/ (accessed on 29 January 2021). In the Table S1 details of the Ti compounds simulated is provided. The XANES simulations from this source are performed using FEFF XANES code [42].

### 2.4. Transmission Electron Microscopy (TEM)

To evaluate TiO_2_ particles size and shape by TEM, aqueous suspensions were prepared. One µl was placed on TEM grids covered with formvar and allowed to settle. The grids were then stained with uranyl acetate, washed, and left to dry. In addition, small cubes of control and treated roots, taken in the same area sampled for the SR based μXRF and µXANES studies, were pre-fixed in Karnovsky solution [43], post-fixed in osmium tetroxide, dehydrated, and embedded in Epon 812-Araldite A/M mixture. Thin cross-sections were stained with uranyl acetate and lead citrate. All samples were observed under a FEI Tecnai G2 Spirit electron microscope at 100 kV. ImageJ software was used for the elaboration of TEM micrograph for particles characterization.

### 2.5. Statistical Analysis

To achieve normality, data of Ti concentration in the roots and the shoots of *P. sativum* plants were firstly log10 transformed. Means of four replicates’ analyses were compared using one-way ANOVA and significant differences were identified applying the post-doc Tukey test. Differences were considered significant at *p* value < 0.05. Principal component analysis (PCA) was performed on second derivative data of µXANES spectra using the Savizky-Golay algorithm with 11 points filter and second polynomial order, the data was then treated by vector normalization. The four main groups of Ti-containing compounds, obtained from PCA, were then validated by a logistic regression and least absolute shrinkage as selection operator (LASSO) using a three-fold cross-validation method. The resulting model’s robustness parameters area under curve (0.989), classification accuracy (0.966), precision (0.966), and sensitivity (0.966) values are considered optimal according to Gautam et al. (2015) [44]. In Table S2, the classification results from the logistic regression are presented in a confusion matrix.

## 3. Results

### 3.1. TEM Observations

**Figure 1:**
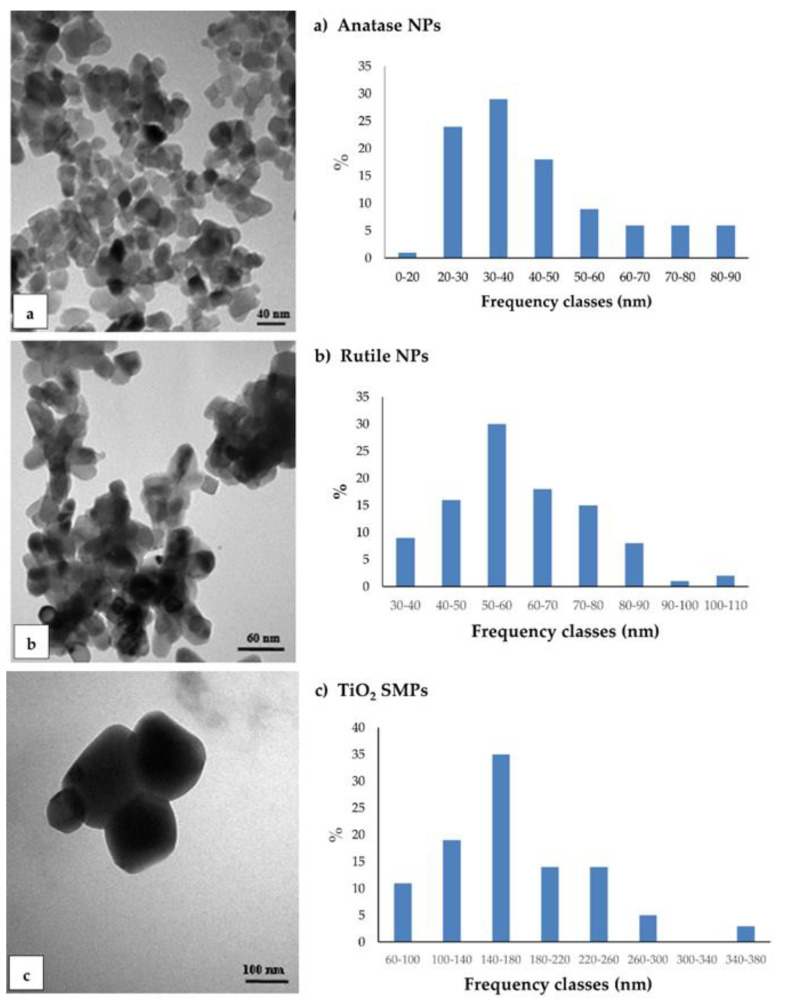
Transmission electron microscopy (TEM) observation and size distribution of TiO_2_ particles grouped in frequency classes for the major axis: (**a**) anatase nanoparticles (NPs); (**b**) rutile NPs; (**c**) TiO_2_ submicron particles (SMPs). ImageJ program was used for TEM images elaboration at sizes measurement.

TEM observations were conducted to characterize the NPs and to detect their presence, localization, and changes in ultrastructure root cells. Under TEM, commercial anatase and rutile NPs and TiO_2_ SMPs appeared highly aggregated, differing in size and shape (Figure 1). Anatase NPs showed little differences in the length of axes, then were considered almost spherical. The axis size varied from about 15 nm to 90 nm with predominant sizes between 30–40 nm (about 30%) (Figure 1a). Rutile NPs showed a rod-like shape with cusps. Since the size of minor axis was nearly constant (about 20 nm), only the major one was considered; major axis varied from about 30 to 110 nm with predominant frequency classes between 50–60 nm (30%) (Figure 1b). Submicron particles were predominantly spherical in shape with sizes varying from 60 nm to 380 nm, with the predominant class between 140–180 nm (35%) (Figure 1c).

**Figure 2:**
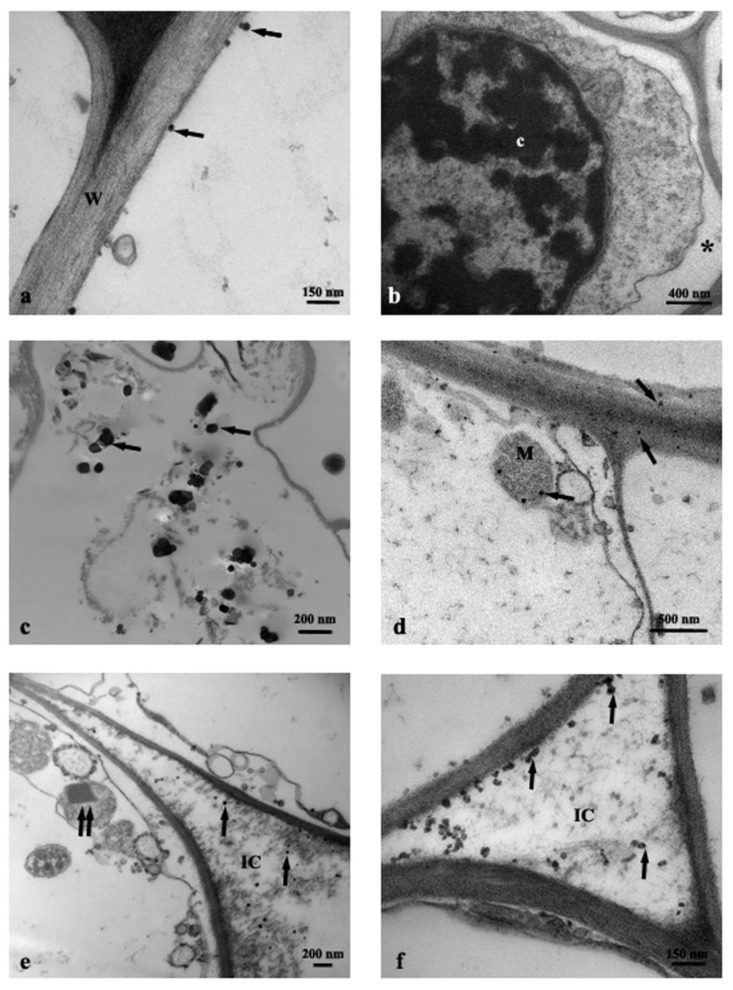
TEM images of roots cross-section: (**a**) C, control, NPs (arrows); (**b**) C, control, plasmalemma-wall detachment (asterisk); (**c**–**f**) Ana800 treatment, NPs (arrows), a crystal in a mitochondrion (double arrow). c, chromatin; IC, intercellular space; M, mitochondrion; W, cell wall.

**Figure 3:**
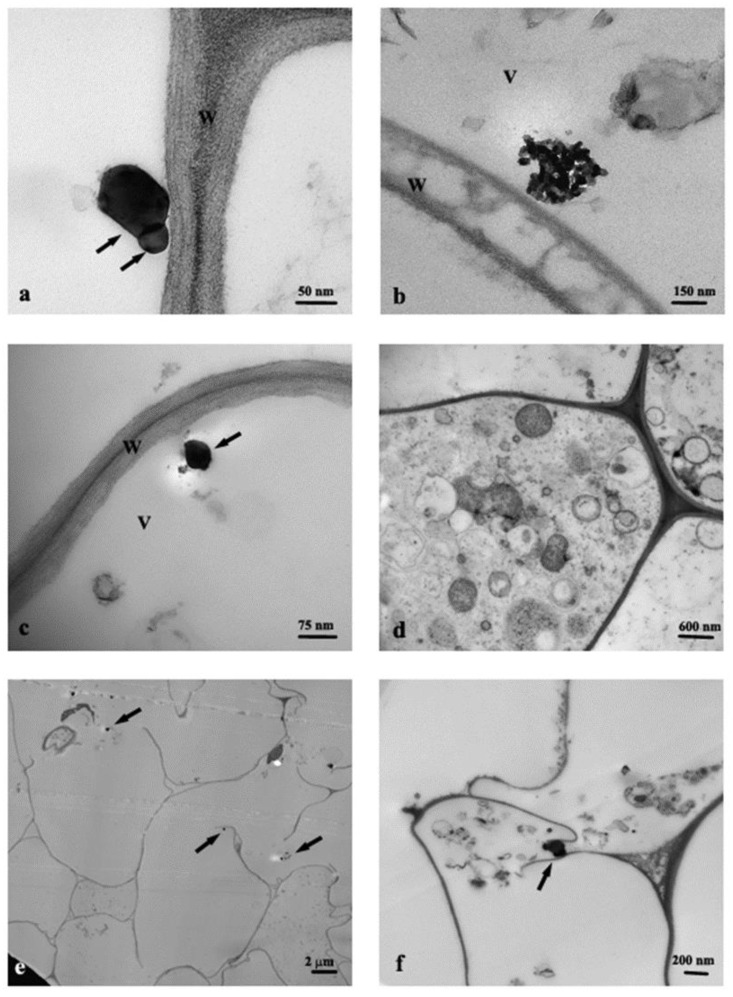
TEM images of roots cross-section: (**a**) Ana800 treatment, NPs in a vessel (arrows); (**b**) Rut800 treatment, aggregate with rutile NPs; (**c**,**d**) Mix800 treatment, cells with cytoplasm in degeneration state; (**e**) SMP800 treatment, NPs (arrows); (**f**) Ana800 treatment, NPs (arrows) crossing ruptures of wall and plasmalemma of adjacent cells. V, vacuole; W, cell wall.

Cross-section of control roots (C) observed by TEM showed the presence of round profile NPs of about 30–50 nm, resembling in shape and size the anatase crystalline phase, and present in the cortex cells, prevalently near the rhizodermis (Figure 2a). Moreover, the cells from C roots often showed evident plasmalemma-wall detachment as in plasmolysis, nuclei with condensed chromatin, and mitochondria with swollen cristae denoting a state of cellular distress (Figure 2b). Root cells from Ana800 treatment showed approximately round-shaped NPs, large (60–100 nm) and small (15–50), isolated or in form of small aggregates of about 3–6 NPs (Figure 2c–f). Small NPs were observed inside the mitochondria, crossing the cell wall, or in intercellular spaces (Figure 2d–f). Large NPs were present in vacuoles (Figure 2c), in cytoplasm, and in vessels (Figure 3a). NPs were mainly evident and numerous in cortex cells near the rhizodermis and hardly detectable in the cortex cells neighboring the endodermis or in central cylinder cells. In Rut800 treated root cells, NPs were prevalently detected in form of large aggregates in vacuoles of cortex cells near rhizodermis (Figure 3b). In the roots from Mix800 treatment, electron-dense particles, prevalently round shaped, resembling anatase NPs, isolated or in form of small aggregates, were observed, mainly in cells close to rhizodermis as in the Ana800 samples (Figure 3c). Finally, in the roots from SMP800, the cells were mostly empty or showed degenerated cytoplasm. Large nanoparticles and particles up to 300 nm in size, isolated or in aggregates of few particles were present, mainly in cortex cells near to rhizodermis. Cell ultrastructure of all treated roots showed similar alterations, in particular plasmolysis at different stages and mitochondria with swollen cristae and crystals, cytoplasm often in a state of degeneration (Figure 2e and Figure 3d). Moreover, in all treated roots, mainly in correspondence of the cells of the cortex, closest to the rhizodermis, where the NPs were more concentrated, it was possible to observe breaks of the wall and therefore of the plasmalemma. Through these ruptures, rarely reported before, cell organelles, different cytoplasmic material, and NPs appeared to transfer from one cell to the adjacent one (Figure 3e,f). Among all treated samples, Ana800 and Rut800 showed more severe damages.

### 3.2. Synchrotron Radiation Based µXRF and µXANES

**Figure 4:**
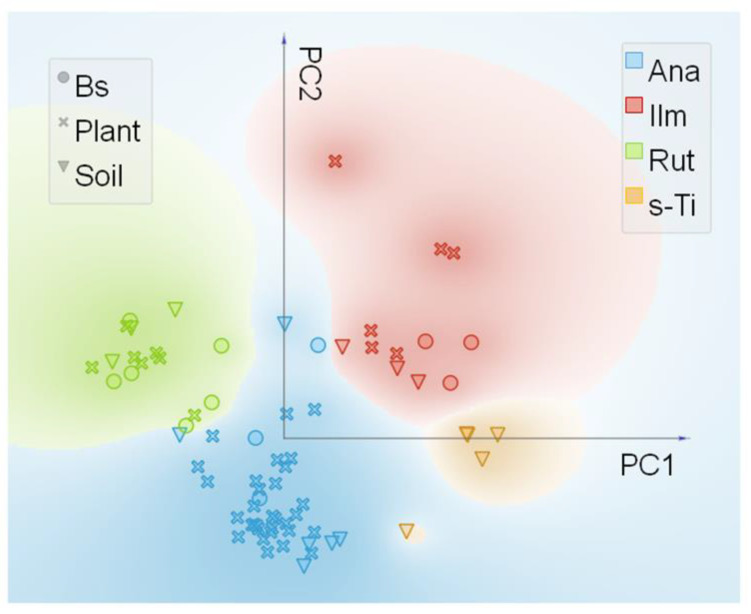
Principal component analysis (PCA) and pre-edge micro X-ray Absorption Near-Edge Structure (µXANES) scatter plot of PC1 vs. PC2 from the experimental µXANES spectra obtained from all plant treatments, biosolid, and soil samples. The colors were attributed to the four main groups achieved and named as Ana (anatase like), Ilm (ilmenite like), Rut (rutile like), and s-Ti (Ti-containing compounds from soil).

**Figure 5:**
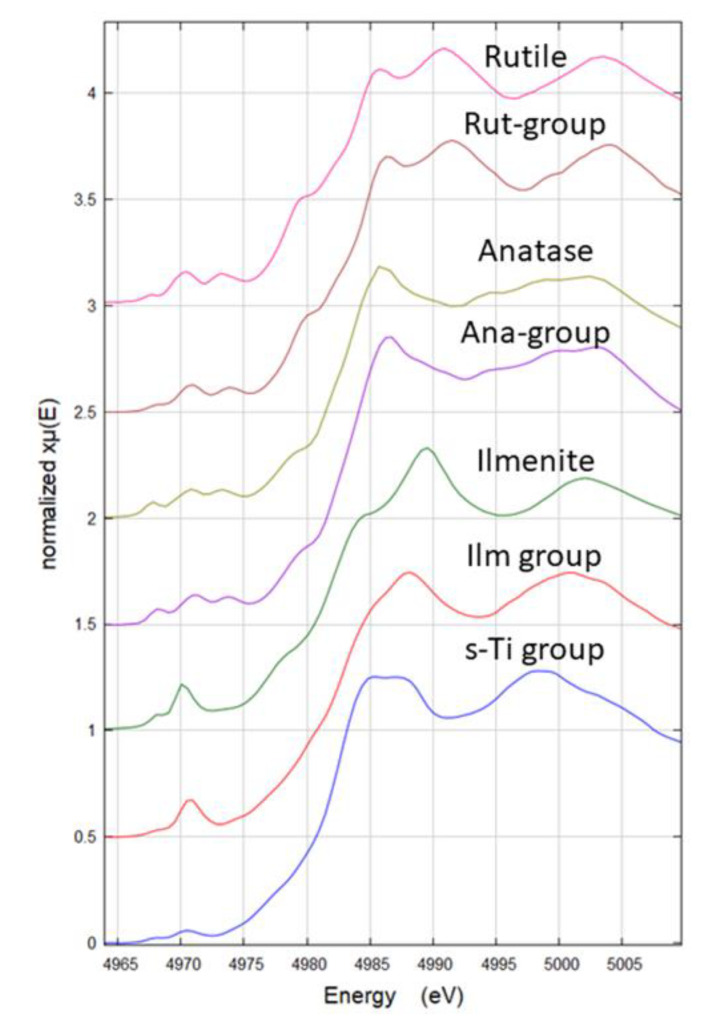
Experimental micro X-ray Absorption Near-Edge Structure (µXANES) spectra of each group from the PCA analysis (Rut-group, Ana-group, Ilm-group, and s-Ti group) and the corresponding µXANES spectra of references (rutile, anatase, ilmenite). s-Ti group was compared with the theoretical spectra of compounds shown in Figure S1 and Table S1. The XANES spectra named ‘group’ are the average of all instances in each group.

Synchrotron radiation based µXRF and µXANES techniques were used to outline the differences in phase composition in all samples and the relative preferential uptake of TiO_2_ particles in the roots. Ti elemental maps (µXRF) on the samples of soil, biosolid, and all plant treatments (C, Ana800, Rut800, Mix800, SMP800) (Figure S1) showed the selected rich-dense Ti spots, in which µXANES spectra were obtained. All spectra taken in the selected spots were arranged in a database, and a subsequent analysis by PCA with an unsupervised statistical routine, was performed. It allowed to draw groups in the data, based on their spectral similarities. Figure 4 showed the four groups obtained from the PCA analysis. After validation of the created groups, using a logistic regression model, the spectra from each group were averaged for comparison to experimental references (Figure 5). Pure anatase and rutile TiO_2_ NPs utilized for plants exposure were used as experimental references, as well as ilmenite (a titanium-iron oxide) and the comparison with the theoretical XANES spectra, which permitted the assignment of the polymorphs in all samples. 

The four groups distinguished by PCA analysis were named based on the observation of the main spectral features. Ana- and Rut- groups were named based on their similarity to experimental references. The group Ilm was named based on the similarity to the experimental reference FeTiO_3_, which shows pre-edge features consistent with experimental and theoretical data from Phoohinkong et al. (2020) [45]. Regarding the last Ti species group, any of the used experimental references showed spectral features comparable, this species was present only in the soil sample (Figure 4 and Figure 5) and was then named s-Ti group, a Ti-containing compound from soil.

To elucidate the structural nature of this last species (s-Ti group), a comparison with theoretical XANES spectra was done. In Table S1 a list of all Ti compounds used to make distinction between four-fold, five-fold, and six-fold coordinate Ti systems. The XANES theoretical spectra from several compounds having four-fold (4c), five-fold (5c), five–six-fold (5–6c), and six-fold (6c) Ti coordination were averaged to provide a reference spectrum to use as guide for the pre-peak interpretation. In Figure S2a, the pre-peaks of the theoretical and experimental Ti XANES are shown. In this figure it is possible to see that 4c Ti species have the highest intensity pre-peak and are shifted to lower energies (4969.7 eV), instead, slightly shifted to higher energies (4970 eV) and less intense pre-peak are the 5c Ti species. The mixed 5–6-c and the 6-c Ti species are shifted +1.3 eV respect to the 4-c and less intense, with 6-c Ti presenting the least intense pre-peak. The experimental s-Ti group presented the least intense pre-peak of all experimental spectra and is in the energy range of the 6-c and 5–6-c species, indicating that s-Ti species is not a 4-c Ti species. These results agree with Ti pre-peak analysis reported in the literature on theoretical and experimental XANES [46]. However, a precise attribution of mineral phase or coordination number was not possible, but the results suggested that s-Ti is a six-coordinate Ti species with a cubic crystal system (Figure S2b). Hence, these results explained the reason why this species was only found in the soil and suggest this is not a main source of Ti to the plants as are the other three Ti species identified. 

From the µXANES spectra in the four groups a % frequency of occurrence of the identified Ti species was obtained for each of the treatments (Table 1). The results from soil and biosolid without plants showed that Ti is present as mix of anatase, rutile, and ilmenite like Ti species and only in the soil the species named s-Ti was observed. In the soil, anatase was the dominant TiO_2_ phase, being about 35% of the species found, while in the biosolid rutile was the most frequent species found, being 50% of the species identified in Bs (Table 1).

Regarding the roots samples, in control roots (C) anatase was identified as the dominant crystalline phase, which represented about 83% of the spots investigated, followed by ilmenite (16.7%) (Table 1). Spots of Ti rich areas in control were frequently localized in the epidermis and in the cortex (Figure S1C), corroborating with the TEM localization. Similarly, all roots from the treated Bs-amended soils showed Ti rich areas more localized near the root epidermis and cortex, few were observed in vessel (Figure S1D–H). Moreover, µXANES spectra in the roots Ana800 indicated that about 63% of the spots investigated were attributed to the anatase phase, followed by ilmenite (22.7%) and rutile (13.6%) (Table 1). Interesting to note is that in the Rut800 roots both the anatase and the rutile crystalline phases were identified with low predominance of the rutile respect to the anatase (57 and 43%, respectively) (Table 1). In addition, the results from the exploitable µXANES spots in the roots Mix800 and SMP800 showed anatase as a dominant phase (100%) (Table 1), although the reason of this unique trend was perhaps influenced by the low number of spectra in both samples.

### 3.3. Uptake of Ti by Roots and Its Translocation to Shoots

*Pisum sativum* plants growing under the TiO_2_ treatments on Bs-amended soil presented differences on Ti accumulation in both roots and aerial parts, when compared to control plants. Control plants showed a relatively high Ti content in the roots (about 420 mg kg^−1^) and limited transport to the shoots (TF = 13 × 10^−3^) (Table 2). The presence of Ti was confirmed in numerous plants species grown in soils without Ti or TiO_2_ addition [47]. However, for all TiO_2_ treatments, Ti accumulation in the roots increased, respect to the control, from 38% to 56% regardless the crystalline phase or the particle size. In addition, the TF also increased for all treatments, and was particularly higher at the treatments with NPs, more precisely, TF increased from 261% to 315%, for Ana800 and Rut800 treatments, followed by Mix800 (192%), and SMP800 (84.6%), respect to the control (Table 2). Besides the augmented Ti translocation for the treatments with NPs, respect to control, all plants showed a general limited transfer to the shoots, demonstrated by the very low values of TF, which found agreement with the observations by TEM and the µXRF data. In fact, the used image-based techniques evidenced a gradient of NPs’ presence from cell wall to cortex and vessel. 

## 4. Discussion

In view of increasing uses and production of TiO_2_ NPs and yet unknown consequences due to their transfer to plants and food chain, the study focused on the investigation of the effects of TiO_2_ NPs on the roots of *P. sativum* L. plants. Roots are the organ/tissue with direct contact with the engineered NPs in a biosolid-amended soil. Biosolid, although being an important amendment for soil quality improvement, is nowadays a vehicle of NPs spread in soil to food plants. Image-based techniques and quantitative evaluation of Ti uptake were used for the investigation of the effects of different crystalline phases and sizes as well as for the localization and identification of TiO_2_ polymorphs. 

The observation by TEM of both pristine NPs and roots cross-section permitted to identify the shape and size of electron-dense particles or their aggregates inside the roots. Aggregates and single NPs were observed in the control roots, which showed the predominant presence of anatase NPs and evident cells’ ultrastructure damages. Anatase NPs in the control roots should come from TiO_2_ particles already present in the original soil and/or the Bs, and their presence in the control roots could be one of the reasons for cell ultrastructure distress. However, it should not be excluded the eventual presence of phytotoxic elements/compounds in the Bs, among them the highly available Cu and Zn evidenced in the same Bs [20] or the presence of non-humified toxic organic compounds (e.g., phenols and ammonia) resultant from a non-complete mineralization of organic matter in Bs [48].

In addition, *P. sativum* plants growing in the TiO_2_ treated Bs-amended soil evidenced increased Ti roots uptake, which caused severe damages in root cells ultrastructure demonstrating that the accumulation of nano and submicron particles in agricultural soil induces higher entrance of TiO_2_ particles in the roots.

Several studies reported that different NPs, mainly with dimension smaller than cell wall pores (about 20 nm) or their aggregates, were present in the apoplastic spaces [49]. These studies suggested an apoplastic pathway for NPs transport in plant roots, through which they can cross the cell wall passing between the pores itself or causing a loosening in the wall mesh [49]. In fact, in our work only the small NPs were observed crossing the cell wall and in intercellular spaces. By this mechanism, NPs can cross the cell walls of epidermis, those of parenchymatic cortical cells and reach the endodermis, where the Casparian strip may prevent their entry in the vascular cylinder [50]. However, NPs may be able to overcome this barrier by entering in the xylematic flow in the root tip region, where the Casparian strip has not yet formed or through the lateral root junction, where the Casparian strip is disconnected [51]. Indeed, in our treated roots rare NPs, also of large size, were observed in the vessels. Another hypothesis for NPs pathway through root cells is the symplastic route, which is based on the passage from one cell to the other through the plasmodesmata, or by NPs penetration by cell membrane [51]. It was supposed also that the NPs caused the local production of ROS, which would lead to the degradation of structures such as cell membrane and cell wall [49]. The increased ROS production in *P. sativum* roots was evidenced in our previous study through the observation of increased levels of H_2_O_2_ and lipid peroxidation, in particular for the anatase and the mix of anatase and rutile treatments, that confirms the hypothesis described above [18]. On this basis, in the present study, the ruptures observed in the cell walls and plasmalemmas could be caused by the presence of NPs, allowing larger ones to pass from one cell to another, either isolated or in small aggregates, thus evidencing another possible path of intercellular translocation. All these mechanisms may rule the movement of TiO_2_ NPs in the pea roots. In addition to the movement of NPs in the roots, some long-distance movement was supposed with the Ti elemental analysis, which showed a marked increase of TF in respect to control plants and in particular for the treatments with NPs.

Roots are able to uptake the different Ti species spiked or already present in the growth matrix, where SR based µXRF and µXANES techniques provided information on the localization of the element in the roots cross-section by mapping it and allowing the species identification by the Ti K-edge µXANES spectra. This last is a useful fingerprint tool for the identification of TiO_2_ phase composition where the pre-peak region allows to categorize the coordination structure of Ti species [46,52].

Titanium elemental maps on the soil and the Bs samples revealed considerable occurrence of Ti agglomerates or Ti rich areas. The presence of anatase, rutile, and ilmenite, and the other Ti-containing mineral (e.g., s-Ti), identified by µXANES spectra in the original soil, is foreseeable, since the occurrence of natural Ti background is relatively high, accounting for about 0.6% in European soils. Generally, rutile is reported as one of the most common TiO_2_-minerals, anatase being distinguished less frequently, and ilmenite the major titanium-iron oxide mineral [53]. Recent studies also identified the presence of TiO_2_ nano-forms and colloidal Ti-containing particles in soil and sewage sludge [54,55,56,57]. The presence and predominance of rutile over anatase in our Bs (50% vs. 25%) could be mainly attributed to their presence as engineered nanoparticles in consumer products (personal care products, food grade additive in foods and drugs, coatings, paints and pigments, etc.), that will end up in WWTPs, increasing the local fluxes of TiO_2_ [54,58]. For example, Tong et al. (2014) [57] found a clear predominance of rutile over anatase in the Bs from Illinois, USA (60% vs. 30%), similar to our results with Bs from Pisa, Italy, while the study of Pradas Del Real et al. (2018) [56] found a reversed predominance but with lower difference between the two forms in the Bs from Dubendorf, Switzerland (45% vs. 55%). On the other hand, the higher presence of anatase (about 35%) over the other three Ti polymorphs found in our soil may probably reflect both geological/weathering processes and anthropogenic sources. Therefore, the presence of natural and/or anthropogenic Ti compounds identified in the soil and the Bs probably reflect the TiO_2_ polymorphs taken up and identified in the pea roots, since TiO_2_ is considered chemically stable and hard to produce ions by dissolution [59]. Results of SR based µXRF and µXANES analysis performed in the roots, in fact, identified the same crystalline phases found in soil or Bs. 

Synchrotron radiation spectroscopic analysis on the control roots suggested that anatase (and ilmenite) come mainly from the soil, since anatase was the most abundant phase found in the original soil (35.3%) but was one of the less frequent Ti species in the Bs (25%). In addition, being the Bs amended in the soil at about 3% (DW basis), the contribution of anatase from the Bs in the control Bs-amended soil should be about 0.06%. Instead, treated roots indicated a facilitated uptake of anatase, respect to the other polymorphs. This was clearly demonstrated in the PCA analysis, which showed that Ana-group, respect to the other groups, was highly represented by the plant samples. In fact, in the roots from Rut800 treatment, anatase represented more than 40% of Ti species, while in the roots from Ana800 treatment it represented 63%. Moreover, despite the restricted number of spectra in both the Mix800 and the SMP800 roots, the dominance of anatase phase in these samples find agreement with TEM observations, which detected in those treated roots only particles resembling the round anatase shape. Both µXRF and TEM analysis in the pea roots, identified a gradient of Ti particles localization from the cortex cells near the rhizodermis to the endodermis and the central cylinder cells. The preferential localization of NPs near the rhizodermis and the low TF in all treated plants is probably related to low intercellular mobility of NPs in *P. sativum* plants. The low translocation to shoots could end up with a limited entry of NPs in the edible part of the plant. 

This low translocation did not permit the Ti species identification and localization by SR spectroscopic techniques. These results found accordance with other studies that have proven the transfer of TiO_2_ NPs from soil to root and edible parts of plants [60,61,62], although few studies were focused on plants growing in Bs-amended soil [28]. For instance, the study of Servin et al. (2013) [60], using μ-XRF and μ-XANES techniques, identified the root-to-fruit translocation and both crystalline phases in the fruits of *Cucumis sativus* L. plants growing in soil spiked with TiO_2_ NPs (mix of 80% anatase and 20% rutile). In addition, alteration of nutritional values and quality of fruits were proved by the decomposition of nutritional macromolecules, the increase of catalase activity and the decrease of ascorbate peroxidase activity in function of TiO_2_ concentration in soil [60].

In our study, the severe ultrastructural damages of the treated root cells indicated the potential toxicity of TiO_2_ NPs accumulated in Bs. More pronounced damages were observed for the separate anatase and rutile treatments. In addition, the significant presence of anatase crystalline phase in all treated samples suggests a preferential uptake of this polymorph. The prevalence of anatase in the treated roots and the pronounced damages in cells ultrastructure shed new light on our previous studies on *P. sativum*. Anatase NPs induced higher roots reactivity with altered oxidative signaling processes and lower growth, although no clear differences, in respect to the crystalline phases, were found for the NPs’ interference on plant mineral nutrition [18,20]. Taken together, results confirm the potential harm of TiO_2_ NPs in this food crop, however, the full life cycle of plants should be further investigated to evidence the impact of TiO_2_ NPs in edible parts. The low TF found in pea plants grown for 30 days could change as the full life cycle is reached and the effects or the mere presence of NPs in eatable plant tissues may be a cause of danger.

## 5. Conclusions

The present study used image-based techniques and quantitative evaluation of Ti uptake to identify the preferential crystalline phase uptake, its sizes, and localization in the roots of *Pisum sativum* plants, and the Ti distribution between roots and shoots. 

This study contributed to the actual interest and debate on the possible advantages or toxicity of TiO_2_ in agricultural applications and evidenced that the biosolids can be a vehicle of nano and submicron TiO_2_ particles entry and accumulation in plants growing in Bs-amended farm soil. In particular *P. sativum* plants exposed to treated soils showed a preferential uptake of anatase NPs, mainly localized in the root cortex cells, near the rhizodermis, that induced alterations in cells ultrastructure characterized by cytoplasmic and mitochondria damages and plasmolysis.

In conclusion, results from the present study pose an alert for the presence of TiO_2_ nano or larger particles in Bs for agricultural use, in view of use without considering the risk of anthropogenic nanoTiO_2_ accumulation and unknown consequences for soil and food quality. For this reason, more studies should be carried out with TiO_2_ NPs, considering the full plant life cycle, the crop productivity and quality, as well as the transgenerational effects. To limit the exposure of agricultural soils and food crops, an appropriate monitoring and regulation of TiO_2_ NPs content in Bs for use in agriculture is suggested, similarly to other potentially toxic elements.

## Figures and Tables

**Table 1:** Total number of µXANES spectra and % frequency of identified Ti species in the soil, biosolid, and root samples.

	% Frequency of Ti Species Identified				
Treatments	Ilm-Group	Rut-Group	Ana-Group	s-Ti	Total n. Spectra
**SOLIDS**					
soil	17.6	17.6	35.3	29.4	17
Bs	25.0	50.0	25.0	0.0	12
**ROOTS**					
C	16.6	0.0	83.3	0.0	6
Ana800	22.7	13.6	63.6	0.0	22
Rut800	0.0	57.1	42.8	0.0	7
Mix800	0.0	0.0	100	0.0	4
SMP800	0.0	0.0	100	0.0	5

Bs = biosolid; C = Bs-amended soil control; Ana800 = anatase NPs; Rut800 = rutile NPs; Mix800 = mixture of anatase and rutile, 1:1 ratio; SMP800 = submicron TiO_2_ particles; 800 = TiO_2_ dose (mg/kg) in Bs-amended soil.

**Table 2:** Titanium concentration in the roots and Ti translocation factor (TF, ratio Ti shoots to roots) of *P. sativum* plants exposed to different soil treatments. Values are means ± sd (n = 4), different letters in the same column represent significant differences among the mean values (*p* < 0.05).

Soil Treatments	Ti Roots (mg kg^−1^)	TF (Ti _shoot/_Ti _root_) × 10^−3^
C	420 ± 60.5 a	13 ± 2 a
Ana800	591 ± 9.66 bc	47 ± 8 d
Rut800	655 ± 44.5 c	54 ± 9 d
Mix800	580 ± 70.8 bc	38 ± 6 c
SMP800	657 ± 92.0 c	24 ± 3 b

C = Bs-amended soil control; Ana800 = anatase NPs; Rut800 = rutile NPs; Mix800 = mixture of anatase and rutile, 1:1 ratio; SMP800 = submicron TiO_2_ particles; 800 = TiO_2_ dose (mg/kg) in Bs-amended soil.

## Data Availability

The datasets generated during and/or analysed during the current study are available from the corresponding author on reasonable request.

## Supplementary Materials

The following are available online at https://www.mdpi.com/article/10.3390/nano11040921/s1, Figure S1: Tricolor and heat of µXRF maps showing the Ti distribution in all samples. Arrows indicate points where µXANES were collected and numbers correspond to spectrum number indicated in Table S3. Red squares localize the magnified areas used for Ti spots focalization: (A) soil, (B) Bs, (C) control root, (D,E) Ana800 roots, (F) Rut800 root, (G) Mix800 root, (H) SMP800 root.; Figure S2: Pre-peak spectral from theoretical spectra of (a) Ti sites with different coordination numbers (4c, 5c, 6c, 5–6c); (b) spectral comparison of c-Ti with the theoretical spectra from Ti_4_(FeO_4_)_3_ (mvc-14970, cubic), Ti_3_Fe_3_O (mp-504733, cubic), see Table S1 for more details; Table S1: List of Ti-compounds used to obtain theoretical XANES spectra from the https://materialsproject.org/ (accessed on 29 January 2021). The coordination number (CN) was obtained by observation of the cell unit structures. The ID number can be used to access all the structural information deposited in this data base; Table S2: Confusion matrix from the logistic regression model performed with the experimental data from plants, soils and biosolid. The target category used was created from the PCA groups obtained from the data and named Rut (rutile like), Anatase (anatase like), Ilm (ilmenite like) and s-Ti (a Ti-containing compound from soil); Table S3: List of spectrum numbers used to indicate the locations in the µXRF map (Figure S1). Treatment and phase (according to PCA grouping is included). Missing numbers are from spectra used in the PCA model but specific map location was not possible to be attributed; * indicates spectra from soil sample that was analyzed a second time, the spectra was considered as another point in the sample as this was repeated several hours after the relocation precision is then not precise to µm.
