# Supplementary material for: Synchrotron Radiation Spectroscopy and Transmission Electron Microscopy Techniques to Evaluate TiO2 NPs Incorporation, Speciation, and Impact on Root Cells Ultrastructure of Pisum sativum L. Plants
Source: Nanomaterials (Basel). 2021 Apr 4;11(4):921. doi: 10.3390/nano11040921 (PMC8066591; doi:10.3390/nano11040921)
Supplement: Supplementary file 1 [file nanomaterials-11-00921-s001.pdf]

Supplementary Material

Synchrotron radiation spectroscopy and transmission electron microscopy techniques to evaluate TiO_2 NPs incorporation, speciation and impact on root cells ultrastructure of *Pisum sativum* L. plants

Simonetta Muccifora[1], Hiram Castillo-Michel[2], Francesco Barbieri[1], Lorenza Bellani[1,3], Monica Ruffini Castiglione[4], Carmelina Spanò[4], Ana E. Pradas del Real[2], Lucia Giorgetti[3], Eliana L. Tassi[5*]

[1] Department of Life Sciences, University of Siena, Via A. Moro 2, 53100 Siena, Italy

[2] European Synchrotron Radiation Facility, Beamline ID21, 38100 71 Av. Rue des Martyrs 38000 Grenoble, France;

[3] Department of Biology, University of Pisa, Via Ghini 13, 56126 Pisa, Italy

[4] Institute of Agricultural Biology and Biotechnology, National Research Council, Via Moruzzi 1, 56124 Pisa, Italy

[5] Research Institute on Terrestrial Ecosystems, National Research Council, Via Moruzzi 1, 56124 Pisa, Italy

*corresponding author: elianalanfranca.tassi@cnr.it (Eliana L. Tassi)

number of pages: 09; Figures: 2; Tables: 3

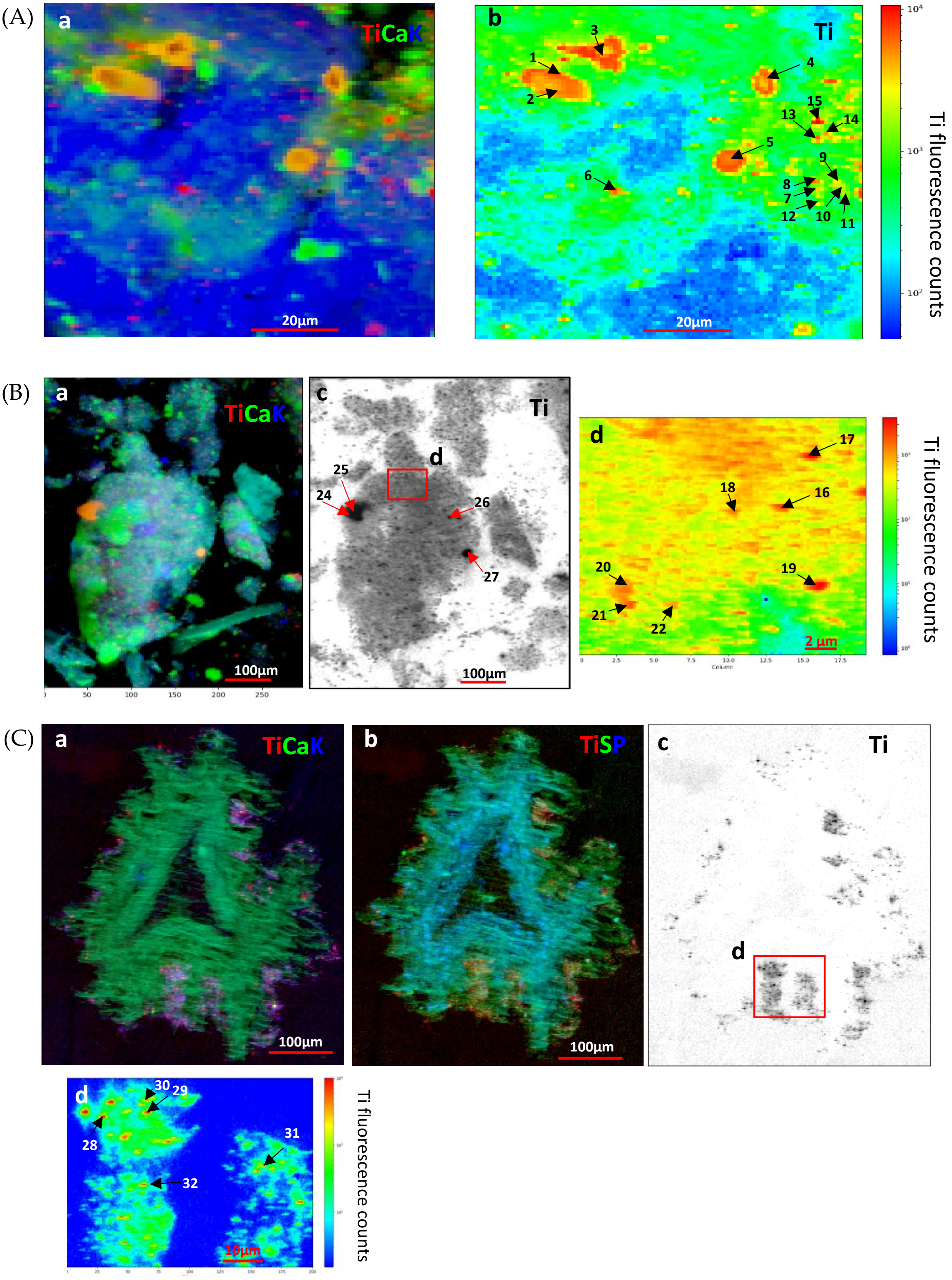

(A)
a
TiCaK
20µm
b
Ti
1
2
3
4
5
6
7
8
9
10
11
12
13
14
15
20µm
Ti fluorescence counts
(B)
a
TiCaK
100µm
c
Ti
d
24
25
26
27
100µm
d
16
17
18
19
20
21
22
2 µm
Ti fluorescence counts
(C)
a
TiCaK
100µm
b
TiSP
100µm
c
Ti
d
d
28
29
30
31
32
10µm
Ti fluorescence counts

(D)
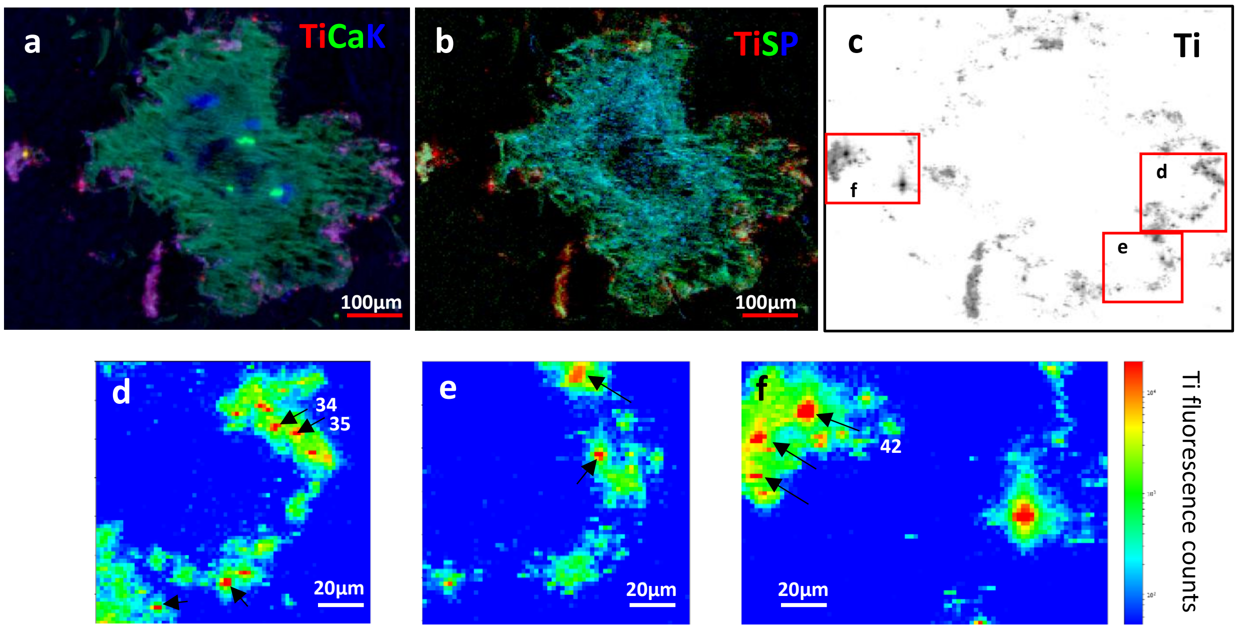
a
TiCaK
100µm
b
TiSP
100µm
c
Ti
f
d
e
d
34
35
20µm
e
20µm
f
42
20µm
Ti fluorescence counts

(E)
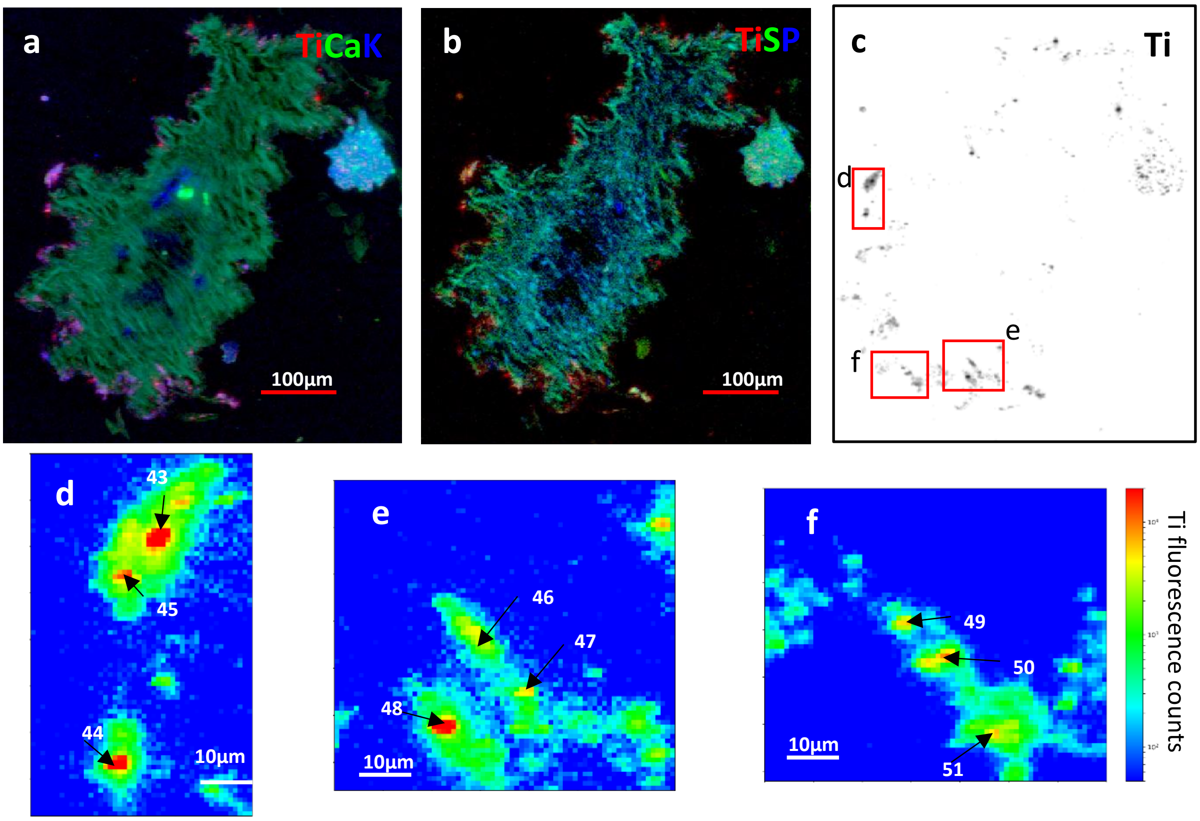
a
TiCaK
100µm
b
TiSP
100µm
c
Ti
d
e
f
d
43
45
44
10µm
e
46
47
48
10µm
f
49
50
51
10µm
Ti fluorescence counts

(F)

(G)

(H)

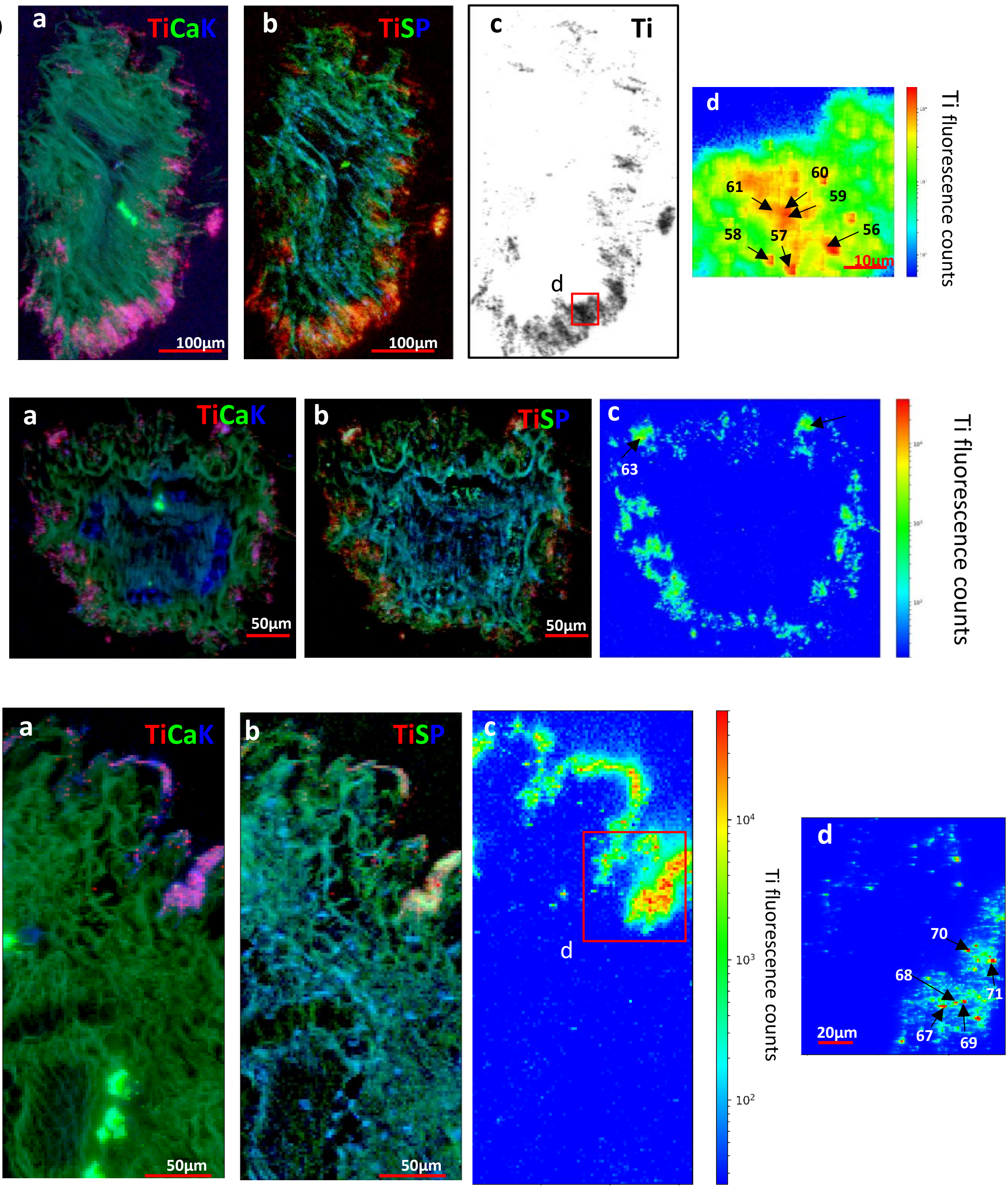

Figure S1 Tricolor and heat of µXRF maps showing the Ti distribution in all samples. Arrows indicate points where µXANES were collected and numbers correspond to spectrum number indicated in Table S3. Red squares localize the magnified areas used for Ti spots focalization: (A) soil, (B) Bs, (C) control root, (D), (E) Ana800 roots, (F) Rut800 root, (G) Mix800 root, (H) SMP800 root.

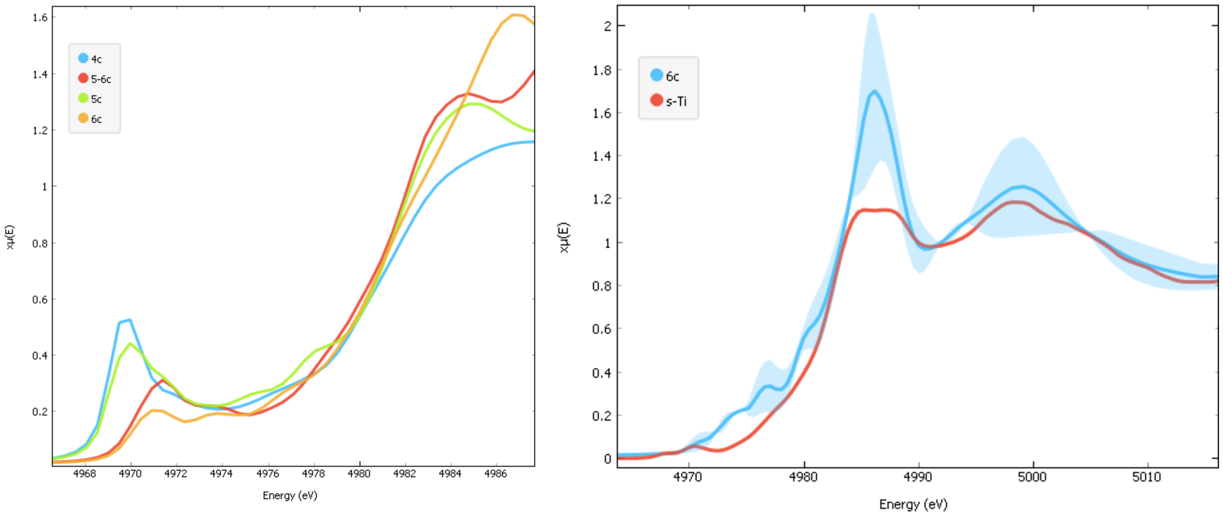

(a) **(b)**

Figure S2 Pre-peak spectral from theoretical spectra of (a) Ti sites with different coordination numbers (4c, 5c, 6c, 5-6c); (b) spectral comparison of c-Ti with the theoretical spectra from $Ti_4(FeO_4)_3$ (mvc-14970, cubic), Ti_3Fe_3O (mp-504733, cubic), see Table S1 for more details.

Table S1 List of Ti-compounds used to obtain theoretical XANES spectra from the https://materialsproject.org/. The coordination number (CN) was obtained by observation of the cell

unit structures. The ID number can be used to access all the structural information deposited in this data base.

Formula	ID	CN	Tag	Point Group	Crystal System
Na_2TiSiO_5	mp-621926	4c	disodiumtecto-titanosilicate	4/m	tetragonal
Ba_2TiO_4	mp-3397	4c	dibarium titanate	2/m	monoclinic
$Na_6Ti_2O_7$	mp-777423	4c	none	2/m	monoclinic
$Na_6Ti_2O_7$	mp-778364	4c	none	2/m	monoclinic
$Na_2Ti_2O_5$	mp-779729	4c	none	mm2	orthorhombic
Ti_2FeO_5	mp-31857	5-6c	iron dititanium oxide	mmm	orthorhombic
$TiFe_2O_5$	mp-24977	5-6c	pseudobrookite	mmm	orthorhombic
$Rb_2Ti_6O_{13}$	mp-540784	5-6c	rubidium 13-oxohexatitanite	2/m	monoclinic
$Sr_4Ti_5(Si_2O_{11})_2$	mp-558553	5-6c	matsubarite	2/m	monoclinic
$Na_2Ti_3O_7$	mp-3488	5-6c	disodium titanate	2/m	monoclinic
Na_2TiSiO_5	mp-6138	5c	natisite	4/mmm	tetragonal
Na_2TiSiO_5	mp-555976	5c	disodium tecto-titanosilicate	mm2	orthorhombic
$Ba_2Ti(SiO_4)_2$	mp-6081	5c	barium titanium silicate	4mm	tetragonal
$K_2Ti_2O_5$	mp-28075	5c	dipotassium dititanium oxide	2/m	monoclinic
Rb_2TiO_3	mp-5403	5c	dirubidium titanate	mmm	orthorhombic
$KNaTiO_3$	mp-560767	5c	potassium sodium titanate	2/m	monoclinic
$Ba_2Ti(GeO_4)_2$	mp-6289	5c	dibarium titanogermanate	4mm	tetragonal
$Na_2Ti_2O_5$	mp-779477	5c	none	2/m	monoclinic
$NaTiO_3$	mp-977123	5c	none	m-3m	cubic
$TiFeO_3$	mp-19270	6c	ilmenite	3m	trigonal
$TiFe_2O_5$	mp-566709	6c	di-iron(III) titanate	2/m	monoclinic
$Ti(FeO_2)_2$	mp-33684	6c	none	mmm	orthorhombic
$TiFe_2O_5$	mp-19255	6c	di-iron(III) titanium oxide	2/m	monoclinic
$TiFeO_3$	mp-25002	6c	iron(III) titanate	mmm	orthorhombic
$TiFeO_3$	mp-19074	6c	iron(III) titanate	mmm	orthorhombic
$Ti_4(FeO_4)_3$	mvc-14970	6c	none	m-3	cubic
$Ti_3Fe_7O_{15}$	mp-34287	6c	none	m	monoclinic
Ti_3Fe_3O	mp-504733	6c	iron titanium oxide	m-3m	cubic
$Ti(FeO_2)_4$	mp-35482	6c	none	2/m	monoclinic
$Ti_2Fe_4O_9$	mp-766757	6c	none	m	monoclinic
$Na_2TiSi_4O_{11}$	mp-556283	6c	sodium titanium silicate	4/m	tetragonal
$Na_2Ti_2Si_2O_9$	mp-5996	6c	ramsayite	mmm	orthorhombic
$NaTi_2O_4$	mp-29356	6c	none	mmm	orthorhombic
$Na_4Ti_5O_{12}$	mp-555678	6c	none	-3	trigonal
TiO_2	mp-390	6c	anatase	4/mmm	tetragonal
TiO_2	mp-2657	6c	rutile	4/mmm	tetragonal
TiO_2	mp-1840	6c	brookite	mmm	orthorhombic

Table S2 Confusion matrix from the logistic regression model performed with the experimental data from plants, soils and biosolid. The target category used was created from the PCA groups obtained from the data and named Rut (rutile like), Ana (anatase like), Ilm (ilmenite like) and s-Ti (Ti-containing compound from soil).

		Predicted				
		**Ana**	**Ilm**	**Rut**	**s-Ti**	sum
**Actual**	**Ana**	38	1	1	0	40
	**Ilm**	0	10	0	2	12
	**Rut**	2	0	14	0	16
	**s-Ti**	1	0	0	4	5
	sum	41	11	15	6	

Table S3 List of spectrum numbers used to indicate the locations in the µXRF map (Figure S1 A-H). Treatment and phase (according to PCA grouping is included). Missing numbers are from spectra used in the PCA model but specific map location was not possible to be attributed; * indicates spectra from soil sample that was analyzed a second time, the spectra was considered as another point in the sample as this was repeated several hours after the relocation precision is then not precise to µm.

Spectrum #	Treatment	Phase	Figure S1
1	soil	s-Ti *	(A)
2	soil	s-Ti *	(A)
3	soil	Ilm	(A)
4	soil	Ilm	(A)
5	soil	s-Ti	(A)
6	soil	Ana	(A)
7	soil	Ana	(A)
8	soil	Ana	(A)
9	soil	Rut	(A)
10	soil	Rut	(A)
11	soil	Rut	(A)
12	soil	Ana	(A)
13	soil	Ana	(A)
14	soil	Ilm	(A)
15	soil	Ana	(A)
16	biosolid	Rut	(B)
17	biosolid	Rut	(B)
18	biosolid	Ana	(B)
19	biosolid	Rut	(B)
20	biosolid	Rut	(B)
21	biosolid	Rut	(B)
22	biosolid	Rut	(B)
23	biosolid	Ana	(B)
24	biosolid	Ilm	(B)
25	biosolid	Ilm	(B)
26	biosolid	Ilm	(B)
27	biosolid	Ilm	(B)
28	C	Ana	(C)
29	C	Ilm	(C)
30	C	Ana	(C)
31	C	Ana	(C)
32	C	Ana	(C)
	C	Ana	(C)
34	Ana800	Ilm	(D)
35	Ana800	Ana	(D)
36	Ana800	Ilm	(D)

37	Ana800	Ana	(D)
38	Ana800	Ilm	(D)
39	Ana800	Ana	(D)
40	Ana800	Rut	(D)
41	Ana800	Ana	(D)
42	Ana800	Ilm	(D)
43	Ana800	Rut	(E)
44	Ana800	Ana	(E)
45	Ana800	Ana	(E)
46	Ana800	Ana	(E)
47	Ana800	Ana	(E)
48	Ana800	Ana	(E)
49	Ana800	Ana	(E)
50	Ana800	Ana	(E)
51	Ana800	Ana	(E)
	Ana800	Ana	(E)
	Ana800	ilm	(E)
	Ana800	Rut	(E)
	Ana800	Ana	(E)
56	Rut800	Ana	(F)
57	Rut800	Ana	(F)
58	Rut800	Ana	(F)
59	Rut800	Rut	(F)
60	Rut800	Rut	(F)
61	Rut800	Rut	(F)
62	Rut800	Rut	(F)
63	Mix800	Ana	(G)
64	Mix800	Ana	(G)
65	Mix800	Ana	(G)
66	Mix800	Ana	(G)
67	SMP800	Ana	(H)
68	SMP800	Ana	(H)
69	SMP800	Ana	(H)
	SMP800	Ana	(H)
71	SMP800	Ana	(H)
